# Supplementary material for: Continuous home monitoring of Parkinson’s disease using inertial sensors: A systematic review
Source: PLoS One. 2021 Feb 4;16(2):e0246528. doi: 10.1371/journal.pone.0246528 (PMC7861548; doi:10.1371/journal.pone.0246528)
Supplement: S1 Table — (DOCX) [file pone.0246528.s001.docx]

| **Level of bias** | **Score** | **Number of**  **publications** | **Publications** |
| --- | --- | --- | --- |
| High | 0-7 | 0 | - |
| Medium | 8 | 4 | [34][47][52][54] |
|  | 9 | 1 | [32] |
|  | 10 | 1 | [31] |
| Low | 11 | 3 | [33][40][46] |
|  | 12 | 5 | [35][38][36][37][41] |
|  | 13 | 10 | [39][42][43][44][45]  [48][49][50][51][53] |

**Risk of bias.**
