# Supplementary material for: Continuous home monitoring of Parkinson’s disease using inertial sensors: A systematic review
Source: PLoS One. 2021 Feb 4;16(2):e0246528. doi: 10.1371/journal.pone.0246528 (PMC7861548; doi:10.1371/journal.pone.0246528)
Supplement: Attachment — Submitted filename: REVISION - Plos One.docx [file pone.0246528.s004.docx]

ABSTRACT

**“Parkinson’s disease (PD) is a progressive neurological disorder of the central nervous system 17 that primarily affects motor functions”** This statement could be modified, since it is acknowledged that PD is not only affecting the motor function, but it also presents a large diversity of non-motor symptoms.

**“In fact, 23 home free-living data collection is more relevant to assessing PD status as it affects the person, 24 capturing motor function during daily living”** This statement is wrongly justified and incorrect.

**“In summary, inertial sensors are capable of gathering data over a long period of 29 time and provide relevant information”** I would argue these are the main conclusions of the literature review performed.

INTRODUCTION

**“affecting approximately 3% of the population over the 39 age of 65 and 10% over the age of 80 [1].”** Consider an update of the reference and a correction of the epidemiology stated

**“The symptoms of PD are multiple, with many being 40 related to motor dysfunction, such as tremor, rigidity, bradykinesia, and to postural instability 41 [2].”** This statement is vague, consider indicating the relationship of the mentioned symptoms to the diagnose of PD and the traditional consideration of PD as a motor disease

**“Tremor is common, typically occurring unilaterally and later becoming bilateral with**

**43 disease progression [2].”**

*Resting tremor: rhythmic oscillatory involuntary movement occurring in approximately 75% of patients with PD. It appears when the affected body part is relaxed and the action of gravitational forces is not counteracted, particularly in distal parts of the extremities. Resting tremor is presented with a frequency between 3 and 6 Hz and a variable amplitude, from less than 1 cm to > 10 cm. At the onset of the disease, patients typically present asymmetrical tremors, which are more prominent in the upper extremities, equally affecting both sides of the body as the disease progresses.*

**“The “on” and “off” 52 phenomenon, in Levodopa-treated patients, describes motor fluctuations that occur as the levels 53 of dopamine in the brain drop, followed by a worsening of the motor function: during the "on" 54 state the symptoms are well managed, while in the "off" state they deteriorate.”** Consider indicate the duration of the on phase and the daily fluctuation effect.

**Line 57 – 69.** Consider indicating the fact that UPDRS is also time demanding, not routinely performed, unreliable and qualitative assessment.

**“Small-size, lightweight, low-power wearable motion sensors may aid in the objective**

**71 assessment of PD motor symptoms.”** Why and how?

**Line 78 – 90**. Please justify this paragraph better with information from the following reference:

*Long-term unsupervised mobility assessment in movement disorders.*

*Warmerdam E, Hausdorff JM, Atrsaei A, Zhou Y, Mirelman A, Aminian K, Espay AJ, Hansen C, Evers LJW, Keller A, Lamoth C, Pilotto A, Rochester L, Schmidt G, Bloem BR, Maetzler W.*

The connection between the need of daily-life monitoring with IMUS and its clinical relevance should be better justified.

METHODS

Synonyms that could have been used for IMU: body-fixed-sensor, inertial measurement unit, sensor, monitor

RESULTS

Consider including a schema (PRISMA guidelines) on the specific exclusion flowchart.

Consider including a graph with the number of articles found for each specific year of the range

**“Data was collected by accelerometers only (in 46.7% and 50% of the studies in the lab**

**165 or at-home, respectively)”** This statement is confusing, were 46.7% of studies using an accelerometer only in the lab, and the 50% only at home?

**“Fourteen works investigated activity 177 monitoring, eight of which focused on walking 178 [24, 26, 29, 30, 35-37, 44], three on turning [31, 33, 41], two on falls [32, 43] and one on 179 physical activity [46].”** Were these articles not focused on monitoring gait impairments?

**“and four on the on/off 181 state”** since on/off status is not a PD symptom, it should be carefully stated in this sentence to avoid confusion

**“Similarly, 218 Del Din et al. (2016) [37] reported PwP walked with slower and shorter steps”** These findings are in opposition to the precedent statement

**“Moreover, less consistent walking patterns with major fluctuation in kinematics and frequency 221 measures compared to healthy subjects were observed”** This information is technically and clinically relevant and should be further developed. Consider referring to the PwP population as the subject of the sentence.

Table 3. Consider including certain technical information regarding the IMU sensors: sampling frequency, range, duration of assessment, etc.

DISCUSSION

**“yet 283 clinical assessments in a clinic setting provide only a partial overview of the disease’s 284 pathological progression [48].”** this is not the main aim of the literature review, nor the most common used of IMUs for PwP monitoring

**“and hence the quality of life of PwP, allowing them to preserve their independence, avoid 288 additional disease complications and reduce personal”** This statement is not carefully stated, nor justified

**“it was also evident that the adoption of 300 ad-hoc hardware has led to the optimization of the on-board algorithms, enhancing the real301 time feedback given to the PwP and/or clinicians”** This cannot be concluded based on the findings of this article

What is the benefit of long term monitoring? Is there any reliability in a longer measurement?

It could be mentioned the “black box” reality on using the software of commercial devices

Consider mentioning confounders and limitations on real-life monitoring of movements

Limitations on the use of questionnaires for real-life activity monitoring should is neither a main conclusion from this literature review. Did this literature review assess any correlation between quantitative measurements and self-reported outcomes?

**“Sample sizes varied considerably among studies and ranged from one 317 [26, 45] to 170 [43] PwP and from one [26] to 172 [43] controls”** What was the range of the larger sample size including PwP? What is the impact of a reduced sample size? Were any statistical analyses performed in these papers to justify the sample size chosen? Were there any clinical limitation?

**“Concerning the devices’ position during activity monitoring (such as walking and**

**321 turning), most of the papers agreed that a site close to the center of mass such as the waist [29, 322 30, 46] and lower back [26, 31, 32, 35-37, 41, 43, 44] was ideal for impaired gait analysis.”** Why? This needs to be biomechanically justified, since it is relevant for clinical implementation of the devices.

**“consequently a sensor attached on the limbs would capture 325 recordings with large variations in gait patterns during activity monitoring.”** This a wrong statement in the current state.

**“manifest differently and on various locations from person to person [2].”** due to laterality?

**“should be always considered 336 to guarantee the long-term acceptability of the system.”** for what purpose? This needs to be developed and justified

In general, this article needs an exhaustive revision, including a better justification of the statements. Moreover, the aim of this review is not well driven.
